# Activity of histone deacetylase inhibitors and an Aurora kinase inhibitor in BCR-ABL-expressing leukemia cells: Combination of HDAC and Aurora inhibitors in BCR-ABL-expressing cells

**DOI:** 10.1186/1475-2867-13-32

**Published:** 2013-04-04

**Authors:** Seiichi Okabe, Tetsuzo Tauchi, Yuko Tanaka, Shinya Kimura, Taira Maekawa, Kazuma Ohyashiki

**Affiliations:** 1 First Department of Internal Medicine, Tokyo Medical University, Tokyo 160-0023, Japan; 2 Division of Hematology, Respiratory Medicine and Oncology, Department of Internal Medicine, Faculty of Medicine, Saga University, Saga, Japan; 3 Department of Transfusion Medicine and Cell Therapy, Kyoto University Hospital, Kyoto, Japan

**Keywords:** HDAC inhibitor, Aurora kinase inhibitor, T315I mutation

## Abstract

**Background:**

The use of imatinib, an ABL tyrosine kinase inhibitor, has led to a dramatic change in the management of BCR-ABL-positive leukemia patients. However, resistance to imatinib mediated by mutations in the BCR-ABL domain has become a major problem in the treatment of these patients.

**Methods:**

In the present study, we examined the activity of histone deacetylase (HDAC) inhibitors in combination with an Aurora kinase inhibitor in BCR-ABL-expressing cells.

**Results:**

We found the HDAC inhibitors vorinostat and/or pracinostat (SB939) induced apoptosis in BCR-ABL-expressing cells. Additionally, HDAC inhibitors reduced levels of Aurora A and B protein. An Aurora kinase inhibitor, tozasertib (VX-680), inhibited growth, promoted pro-apoptotic activity, reduced the phosphorylation of BCR-ABL and Crk-L, and activated caspase-3 and poly (ADP-ribose) polymerase (PARP) in BCR-ABL-positive cells. Moreover, after treatment with tozasertib, HDAC protein expression was decreased. Combination of vorinostat or pracinostat with tozasertib had a synergistic inhibitory effect on the proliferation of T315I cells. Phosphorylation of Crk-L decreased, and PARP activation increased after treatment with vorinostat or pracinostat and tozasertib. Moreover, combination of vorinostat or pracinostat and tozasertib significantly increased the extent of apoptosis in primary chronic myeloid leukemia cells.

**Conclusions:**

This study demonstrated that combination of HDAC and Aurora inhibitors was highly effective against BCR-ABL-expressing cells.

## Background

Chronic myeloid leukemia (CML) is a hematopoietic disorder characterized by unregulated proliferation of predominantly myeloid cells in the bone marrow [1]. BCR-ABL fusion proteins resulting from the chromosomal translocation t(9;22) (the Philadelphia chromosome: Ph) cause CML [2]. BCR-ABL activity leads to uncontrolled cell proliferation, reduced apoptosis, and malignant expansion of hematopoietic stem cell populations. The ABL tyrosine kinase inhibitor (TKI) imatinib has dramatically improved the management and prognosis of patients with CML [3]. However, some patients, particularly those with advanced-phase CML, have developed resistance to imatinib [4]. More than 50 distinct point mutations in the kinase domain of BCR-ABL have been detected in patients with imatinib-resistant CML; point mutations in this domain are the most frequent cause of acquired imatinib resistance in CML patients [5,6]. Second-generation TKIs, such as dasatinib and nilotinib, have shown promising results in imatinib-resistant CML patients, but dasatinib and nilotinib are not effective against CML clones with T315I mutations [7]. Recently, ponatinib (also known as AP24534) was identified as a potent oral tyrosine kinase inhibitor and was shown to block native and mutated BCR-ABL. Ponatinib is highly active in patients with Ph-positive leukemias, including those with BCR-ABL T315I mutations [8]. However, alternative strategies against point mutations (i.e., T315I) within the BCR-ABL kinase domain are still important to improve the prognosis of CML patients.

Histone deacetylases (HDACs) and histone acetyltransferases (HATs) are enzymes that regulate chromatin structure and function [9]. Modification of histones (e.g., via histone acetylation and deacetylation) plays an important role in the regulation of gene expression [10]. Increased expression of HDACs and disrupted activities of HATs have been observed in several tumor types [11]. HDAC inhibitors are emerging as potent antitumor agents that induce cell cycle arrest, differentiation, and apoptosis in many tumor cells of different origins. HDAC inhibitors represent a new and promising class of antitumor drugs [12]. HDAC inhibitors influence gene expression by enhancing histone acetylation. Because HDAC inhibitors regulate many signaling pathways, cotreatment of HDAC inhibitors with molecular targeted drugs, such as Aurora kinase inhibitors, is a promising strategy against many types of tumors.

This study aimed to examine the activity of the HDAC inhibitors vorinostat and pracinostat (SB939) *in vitro*, both alone and in combination with an Aurora kinase inhibitor. This study also explored the molecular mechanisms underlying treatment-related cell growth inhibition and apoptosis in BCR-ABL-expressing cell lines with point mutations. We found that the combination of HDAC and Aurora kinase inhibitors significantly inhibited cell growth in BCR-ABL-expressing cells.

## Results and discussion

### Activity of HDAC inhibitors in BCR-ABL-positive cells

**Figure 1:**
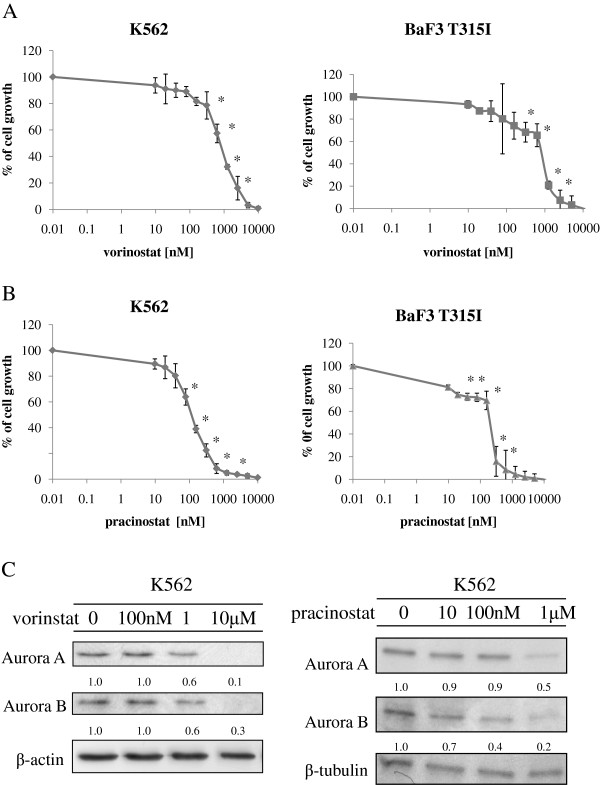
**Effects of HDAC inhibitors on BCR-ABL-expressing cells. **(**A**) K562 and mutant Ba/F3 cells (T315I) were treated with vorinostat or pracinostat for 72 h. The number of viable cells was calculated for each group. *P < 0.05, vorinostat or pracinostat treatment versus control in the same cell line. (**B**) K562 cells were treated with vorinostat or pracinostat at the indicated concentrations for 48 h. Total extracts were analyzed by immunoblotting with anti-Aurora A and B Abs. Actin was used as the loading control. Band intensities were quantified using ImageJ software. Results in A and B are representative of at least 3 reproducible experiments.

HDACs have been identified as novel targets for the treatment of hematologic malignancies, including Ph-positive leukemia. HDACs regulate gene transcription, producing disparate effects on cell growth and survival. Vorinostat, an HDAC inhibitor, was approved by the FDA as therapy for cutaneous T-cell lymphomas. Pracinostat is an oral HDAC inhibitor that is currently in phase II clinical trials [13]. We also reported previously that another HDAC inhibitor, depsipeptide, an acetylated intracellular protein, is effective against BCR-ABL-positive blastic crisis cells [14]. Because vorinostat and other HDAC inhibitors induce cell cycle arrest and apoptosis in tumor cells [15], we investigated whether vorinostat or pracinostat would inhibit growth in BCR-ABL-expressing cells. K562 and Ba/F3 T315I cells were treated with vorinostat or pracinostat, and cell proliferation was investigated. Treatment with vorinostat or pracinostat for 72 h strongly and significantly inhibited the growth of K562 and Ba/F3 T315I cells in a dose-dependent manner (Figure 1A). HDAC inhibitors have been reported to induce the degradation of both Aurora A and B kinases through a proteasome-mediated pathway [16]. Because aberrant expression and activity of Aurora kinases occur in a wide range of human tumors [17], inhibition or depletion of Aurora kinases may provide a promising method to delay the growth of leukemia cells. In this study, we investigated the effects of vorinostat and pracinostat on Aurora kinase expression by using K562 cells. K562 cells were treated with vorinostat or pracinostat at the indicated concentration for 48 h and analyzed by immunoblotting. The expression of Aurora A and B was dose-dependently reduced after treatment with vorinostat or pracinostat (Figure 1B).

### Analysis of the effects of an Aurora kinase inhibitor on intracellular signaling in K562 cells

**Figure 2:**
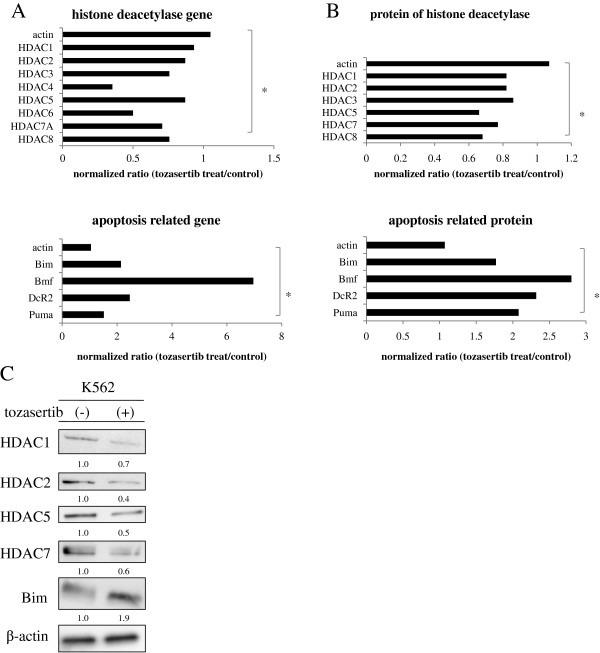
**Analysis of the effects of tozasertib on BCR-ABL-expressing cells. **(**A**) K562 cells were exposed to 1 μM tozasertib or PBS (control) for 16 h. Gene expression data were validated with the Human Genome U133A Genechip. The bar graph shows the expression of HDAC and apoptosis related genes in K562 cells after tozasertib treatment versus control. * P < 0.05 for comparison to the actin expression. (**B, C**) An antibody expression microarray was performed as described in the Materials and Methods section. Protein expression was calculated using MS Excel. Prior to immunoblotting, K562 cells were treated with tozasertib for 24 h. Total extracts were analyzed using anti-HDAC1, -2, -5, -7, and -Bim Abs. Actin was used as the loading control. Band intensities were quantified using ImageJ software. Results in A, B, and C are representative of 3 different experiments. * P < 0.05 for comparison to the actin expression.

Because HDAC proteins are aberrantly expressed in many types of cancers and have nonredundant functions in controlling the hallmark phenotypes of cancer cells [15], we examined HDAC expression after treatment with an Aurora kinase inhibitor in K562 cell lines using DNA and antibody microarray techniques. We found that the relative levels of *HDAC* gene expression in K562 cell lines were decreased after tozasertib treatment. In contrast, expression of apoptosis-related genes, including *Bim*, was increased (Figure 2A). We next examined results of the protein array studies. In K562 cells, we found that HDAC protein levels were decreased and apoptosis-related protein expression was increased after 24 h treatment with 1 μM tozasertib (Figure 2B). To confirm these findings, we performed immunoblotting analysis. Additionally, after tozasertib treatment, the expression of HDAC1, -2, -5, and -7 proteins was significantly reduced, while that of Bim was increased (Figure 2C).

### Activity of the Aurora kinase inhibitor in wild-type and mutant BCR-ABL-expressing cells

**Figure 3:**
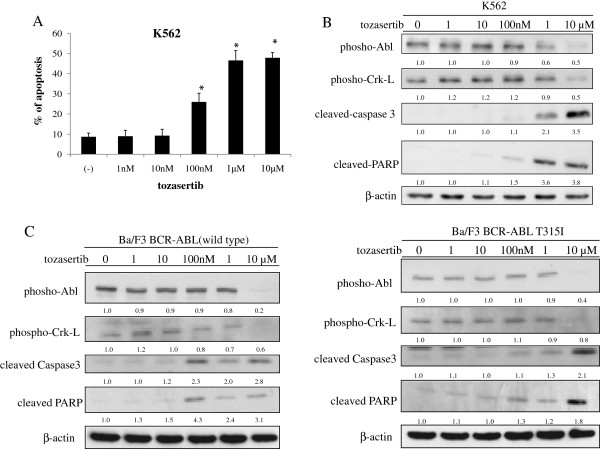
**Effects of tozasertib on BCR-ABL-expressing cells. **(**A**) The percentage of apoptotic cells was estimated by annexin V assay after culturing for 48 h with tozasertib. Results from 3 independent experiments were averaged. Error bars represent standard deviations. *P < 0.05, tozasertib treatment versus control in the same cell line. (**B, C**) K562 and Ba/F3 wt and T315I BCR-ABL cells were treated with tozasertib for 24 h. Total extracts were analyzed by immunoblotting with phospho-specific anti-Abl, -Crk-L, -cleaved caspase 3, and -cleaved PARP Abs. Actin was used as the loading control. Band intensities were quantified using ImageJ software. Data are representative of 3 separate experiments.

We next investigated the activity of tozasertib against wild-type (wt) and mutant BCR-ABL-expressing cells. For this study, we also used Ba/F3 cells expressing wt-BCR-ABL and BCR-ABL with kinase domain mutations found frequently in patients, including T315I. Tozasertib treatment inhibited cell growth in mutant BCR-ABL-expressing cells in a dose-dependent manner “data not shown”. Next, we used flow cytometry with annexin V to examine whether tozasertib could induce apoptosis in BCR-ABL-expressing cells. Tozasertib induced apoptosis in the BCR-ABL-expressing cell line K562 (Figure 3A). We also examined intracellular signaling. The phosphorylation of Abl and Crk-L was decreased after tozasertib treatment (Figure 3B). Caspase-3 and PARP levels were significantly increased (Figure 3B). Similarly, the phosphorylation of Abl and Crk-L was decreased, while caspase-3 and PARP expression levels were increased in BCR-ABL-expressing Ba/F3 cells (wt and T315I; Figure 3C). These results indicated that tozasertib was effective in cell expressing wt-BCR-ABL and BCR-ABL mutants like T315I.

### Efficacy of cotreatment with HDAC and Aurora kinase inhibitors in BCR-ABL-expressing cells

**Figure 4:**
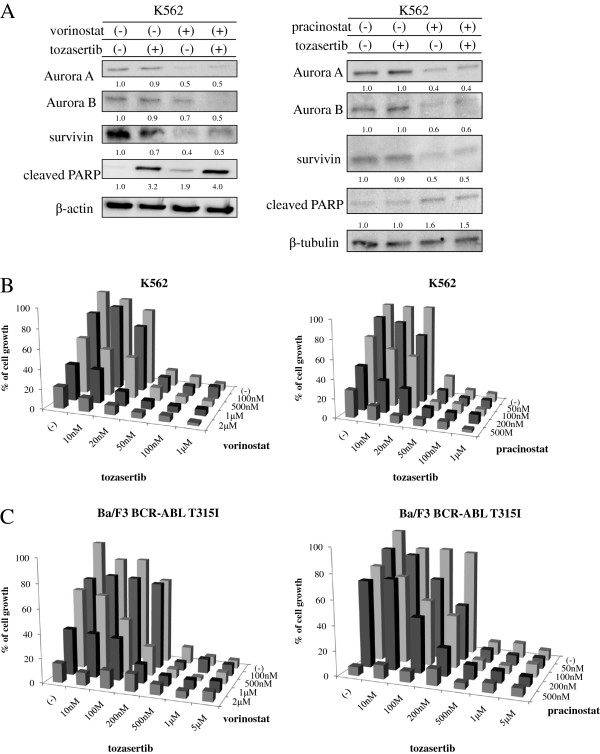
**Effects of vorinostat or pracinostat in combination with tozasertib in BCR-ABL-positive cells. **(**A**) K562 cells were treated with vorinostat or pracinostat and/or tozasertib for 24 h. Total cell lysates were immunoblotted with anti-survivin, -Aurora A and B, and -cleaved PARP Abs. Actin or tubulin was used as the loading control. Band intensities were quantified using ImageJ software. (**B**) K562 cells were treated with HDAC inhibitors (vorinostat or pracinostat) and/or tozasertib for 72 h. The number of viable cells was calculated for each group. (**C**) Ba/F3 T315I cells were treated with HDAC inhibitors (vorinostat or pracinostat) and/or tozasertib for 72 h. The number of viable cells was calculated for each group. Results in A, B, and C are representative of at least 3 reproducible experiments.

Next, we examined the intracellular signaling of HDAC and Aurora kinase inhibitors. The expression of Aurora A and B was reduced after cotreatment with vorinostat or pracinostat and tozasertib. Survivin expression was also decreased, while PARP was activated after cotreatment with vorinostat or pracinostat and tozasertib (Figure 4A). These results suggested that vorinostat or pracinostat affected Aurora kinase expression, while treatment with vorinostat or pracinostat and tozasertib regulated intracellular signaling pathways in BCR-ABL-positive cells. An increased frequency of BCR-ABL point mutations has been found in advanced-phase and recurrent cancers [18,19]. T315I and P-loop mutations, such as G250E, Y253F, and E255K, are highly resistant phenotypes. Next, we investigated whether cotreatment with vorinostat or pracinostat and tozasertib caused growth inhibition in Ba/F3 T315I cells and wt-BCR-ABL-positive K562 cells. Ba/F3 T315I and K562 cells were treated with vorinostat or pracinostat and tozasertib, and cell proliferation was examined. We found that cotreatment with vorinostat or pracinostat and tozasertib significantly inhibited cell growth in both wt-BCR-ABL-positive cells and T315I-positive cells (Figure 4B and C). We also performed statistical analyses to determine the combination index (CI) for vorinostat or pracinostat and tozasertib, which was calculated according to the method of Chou and Talalay [20]. Combination of vorinostat or pracinostat with tozasertib resulted CI values of 0.396 and 0.765. These results suggested that combination of vorinostat or pracinostat with tozasertib synergistically enhanced the toxicities of these drugs in T315I positive Ba/F3 cells. Thus, we demonstrated that tozasertib combined with vorinostat or pracinostat could potentially overcome imatinib-resistance in mutant BCR-ABL-expressing cells. Although high concentrations of compounds were used in these experiments, significantly higher plasma concentrations of these compounds have been reported in clinical trials [21-23]. Additionally, we found that low concentrations of vorinostat or pracinostat and tozasertib were not efficacious in short-term viability assays. However, simultaneous exposure to tozasertib and HDAC inhibitors in long-term survival assays may result in enhanced cell death following treatment with low concentrations of these compounds.

### Efficacy of cotreatment with HDAC and Aurora kinase inhibitors in BCR-ABL-positive primary CML cells

**Figure 5:**
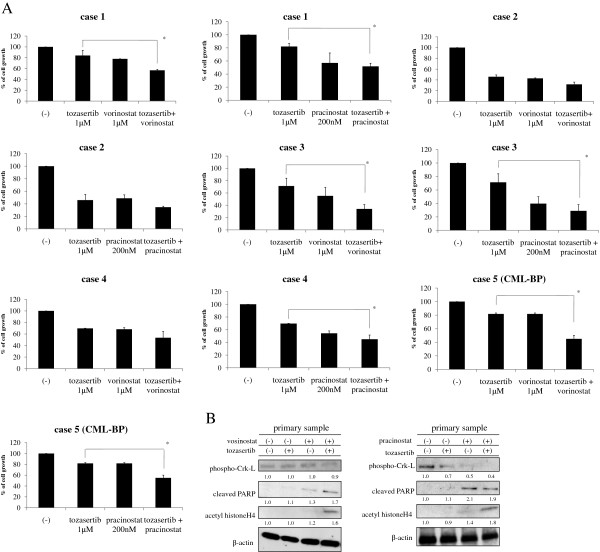
**Effects of HDAC inhibitors and tozasertib on BCR-ABL-positive primary CML cells. **(**A**) Primary CML cell samples and blastic phase samples were cultured at a concentration of 4 × 10^5 ^cells/mL in the presence of HDAC inhibitors (vorinostat or pracinostat) and/or tozasertib for 72 h. The number of viable cells was calculated for each group. *P < 0.05, tozasertib treatment versus tozasertib with HDAC inhibitors in the same line. (**B**) Primary cells were treated with HDAC inhibitors (vorinostat or pracinostat) and/or tozasertib for 24 h. Total extracts were analyzed by immunoblotting with phospho-specific anti-Crk-L, -cleaved PARP, and -acetyl histone H4 Abs. Actin was used as the loading control. Band intensities were quantified using ImageJ software. Results in A and B are representative of 3 different experiments.

Because cotreatment with HDAC and Aurora kinase inhibitors induces significant inhibition of growth in BCR-ABL-expressing cell lines, we next investigated the effects of these compounds in BCR-ABL-positive primary CML samples and blastic phase samples. Indeed, treatment with tozasertib and vorinostat or pracinostat inhibited cell growth in BCR-ABL-positive CML samples and blastic phase samples (Figure 5A). Although we did perform statistical analyses of the data, the sample size was too small to obtain meaningful statistics. Intracellular signaling was also examined. Cotreatment with both tozasertib and vorinostat or pracinostat decreased apparent Crk-L phosphorylation, while apparent PARP and acetyl histone H4 activity was increased (Figure 5B), again indicating the potential efficacy of tozasertib and vorinostat or pracinostat in BCR-ABL-positive primary cells.

## Conclusion

In the current study, HDAC inhibitors induced apoptosis in BCR-ABL-positive leukemia cells. In particular, profound inhibition of cell growth and induction of apoptosis were observed in response to HDAC inhibitors in BCR-ABL-positive K562 and mouse pro-B Ba/F3 cells with ectopic expression of wt and mutant T315I. This response was amplified by cotreatment with an Aurora kinase inhibitor. In this study, we also demonstrated that Aurora kinase proteins were degraded by vorinostat or pracinostat in a dose-dependent manner (Figure 1B). Although the levels of Aurora family proteins were not directly reduced by tozasertib treatment, tozasertib inhibited the expression of HDAC proteins (Figure 2A). As such, our data indicated that vorinostat or pracinostat and tozasertib affected the activities of both Aurora kinase and HDAC, in turn increasing antitumor activity in this system. Clinical trials using tozasertib have been discontinued. However, other pan-Aurora/BCR-ABL dual inhibitors may exhibit a similar profile, and these continue to be studied clinically. Our findings suggest that cotreatment with these compounds and specific molecular-targeted drugs could benefit patients with leukemic BCR-ABL cells that are resistant to more conventional treatments.

## Methods

### Reagents and antibodies

The HDAC inhibitors vorinostat (SAHA, suberoylanilide hydroxamic acid) and pracinostat (SB939) were provided by Selleck Chemicals LLC (Houston, TX). Tozasertib (VX-680, MK-0457) was kindly donated by Vertex Pharmaceuticals Inc (Cambridge, MA). Stock solutions of vorinostat, pracinostat, and tozasertib were dissolved in dimethyl sulfoxide and subsequently diluted to the desired concentration in growth medium. Anti-phospho-Abl, -phospho-Crk-L, -cleaved caspase 3, -PARP-HDAC1, -HDAC2, -HDAC5, -HDAC7, -Bim, and -Aurora A and B antibodies (Abs) were obtained from Cell Signaling Technology (Beverly, MA). Other reagents were obtained from Sigma (St. Louis, MO).

### Cell culture

The human CML cell line K562 was obtained from the American Type Culture Collection (ATCC, Manassas, VA). Ba/F3 wt-BCR-ABL cells and Ba/F3 T315I cells were described previously [24]. These cells were maintained in RPMI1640 medium supplemented with 10% heat-inactivated fetal bovine serum with 1% penicillin/streptomycin in a humidified incubator at 37°C.

### Cell proliferation assay

Cell proliferation analysis was performed as previously described [25].

### Cell signaling assays and western blot analysis

Panorama Ab microarrays (Sigma) were analyzed according to the manufacturer’s instructions. The arrays were scanned using a GenePix Personal 4100A microarray scanner, and normalization was carried out using the housekeeping protein included with the chip. The protein expression ratio was calculated using MS Excel. Western blot analysis was performed as previously described [26,27].

### DNA microarray and microarray data analysis

DNA microarray analysis was performed as previously described [28]. In brief, K562 cells were treated with 1 μM tozasertib for 16 h. Following incubation at 37°C, the cells were washed twice with ice-cold phosphate-buffered saline (PBS) and collected immediately for RNA isolation. In this study, we used the Human Genome U133A Genechip (Affymetrix Santa Clara, CA), which contains more than 47,000 transcripts. Target preparation was carried out following the manufacturer’s expression analysis manual. All arrays were screened for quality by standard methods, and the mean fluorescent intensity for each probe set was determined.

### Primary samples

This study was approved by the Institutional Review Board of Tokyo Medical University, and informed consent was provided by all patients in accordance with the Declaration of Helsinki. Primary samples were obtained from the peripheral blood of CML patients. Mononuclear cells were isolated from blood samples and separated by Lymphosepar (Immuno-Biological Laboratories Co., Gunma, Japan). The cells were cultured in RPMI1640 medium containing 10% fetal calf serum and analyzed as described.

### Flow cytometory analysis

Cells were treated with the indicated concentrations of tozasertib for 48 h. Annexin V/propidium iodide apoptosis assays were performed according to the manufacturer’s instructions (Beckman Coulter, Inc., Brea, CA). The cells were gently mixed and immediately analyzed by flow cytometry.

### Statistical analysis

Differences between treatment groups, in terms of dose response and apoptosis, were determined using Student’s *t* test. *P* values of less than 0.05 were considered significant.

## Competing interests

The authors declare no conflicts of interests.

## Authors’ contributions

SO designed and performed the study, analyzed the data, and wrote the manuscript. TT participated in drafting the manuscript. YT, SK, TM, and KO conceived and designed the study, interpreted the data, and wrote the manuscript. All authors read and approved the final manuscript.
